# Assessment of applicability of Ganga Hospital Score in the management of open tibia fracture

**DOI:** 10.1302/2633-1462.64.BJO-2024-0207.R1

**Published:** 2025-04-21

**Authors:** Innocent Kwizera, Jean C. Byiringiro, J. C. A. Ingabire, Emmanuel Murwanashyaka, Jean L. Mwizerwa

**Affiliations:** 1 College of Medicine and Health Science, University of Rwanda, Kigali, Rwanda; 2 Orthopaedic Department, University Teaching Hospital of Kigali, Kigali, Rwanda; 3 Orthopaedic Department, Rwanda Military Hospital, Kigali, Rwanda

**Keywords:** Amputation, Ganga scoring, Injury severity score, Limb salvage, Open fractures, open tibial fractures, amputations, Soft-tissue reconstruction, infections, Injury Severity Score, wounds, ANOVA, prospective cohort study, tibia, variance

## Abstract

**Aims:**

Open fractures of the tibia encompass a wide spectrum of injuries, posing multiple challenges for treating surgeons. This study evaluates the Ganga Hospital Open Injury Severity Score (GHOISS) in predicting the outcomes of open tibia fractures in Rwanda, focusing on its ability to guide wound management choices and assist in decision-making between preservation and amputation.

**Methods:**

This was a prospective cohort study conducted between March and September 2022 in Kigali, Rwanda, involving patients aged 18 years and older with open tibial fractures. The GHOISS was calculated, and the patients were organized into three groups: Group I: score 1 to 13; Group II: score 14 to 16; and Group III: score ≥ 17. Outcome data were collected at one and six months of follow-up. The predictive validity of the GHOISS was determined through sensitivity, specificity, and predictive values. Correlation and analysis of variance (ANOVA) tests were also conducted to compare groups. Ethical considerations were respected, and institutional review board approval was obtained.

**Results:**

The study involved 111 participants, with a mean age of 34 years (18 to 80) and a male-to-female ratio of 3.44:1. The amputation rate was 10 (9.0%), with a mean hospital stay of 30.55 days (SD 34.09). The infection rate was 54.05%, and the need for soft-tissue reconstruction was 36.9%. The GHOISS in predicting the amputation showed high sensitivity of 100% and sensitivity of 96.03%, with a positive predictive value of 71.4% and negative predictive value of 100%. ANOVA revealed significant differences between the groups (F (2,108) = 21.12; p < 0.001), and a strong positive correlation was found between the covering tissue score and the need for soft-tissue reconstruction.

**Conclusion:**

The GHOISS demonstrated a remarkable ability to predict amputation and salvage in open tibia fractures and the potential for predicting related outcomes. The GHOISS subscore, which assesses skin and covering injuries, has shown a significant ability to predict the need for soft-tissue reconstruction.

Cite this article: *Bone Jt Open* 2025;6(4):463–468.

## Introduction

Lower limb open fractures have remained a global health concern.^1,2^ Globally, there is a scarcity of data reporting their prevalence and incidence. However, studies conducted in higher-income countries (HICs) have estimated the annual incidence to be 30.7 per 100,000 people.^3,4^ They have been reported to occur in younger populations in low-income countries (LICs) compared to HICs.^5,6^ The burden of their complications is common, but was reported to be more significant in LICs.^7^

The management of open tibia fractures poses challenges, especially in LICs.^8^ The majority of Gustilo and Anderson III open fractures require soft-tissue reconstruction procedures, and a delay in open tibia management has been related to an increase in complications, including increased hospital stays, infections, and amputations.^9,10^ The delayed presentation and treatment, unavailability of required implants, and lack of surgeons with the required skills, along with a rising number of road traffic incidents in LICs, were reported to increase the impact of open fractures in LICs.^8,10^

The classification of open tibia fractures remains controversial.^11^ The Gustilo and Anderson classification, developed in 1979, has been dominant in clinical use.^12^ However, some studies, such as Kumar et al,^13^ have shown its weaknesses, including weak reliability, a broad inclusion in Gustilo and Anderson III fractures, inability to predict limb salvage, and not considering other factors impacting outcomes. Several other classifications have been developed, but none have been effective.^14,15^

The Ganga Hospital Open Injury Severity Score (GHOISS) (Table I), developed in 1994 at a major Indian trauma centre, evaluates all aspects of limb injuries, considering comorbidities.^12,16^ Its score has demonstrated remarkable sensitivity and specificity in predicting limb preservation and outcomes, with high accuracy in comparative investigations.^13,16-18^

**Table I.** Ganga Hospital Open Injury Severity Score (GHOISS).

Item	Score	Description
Covering structures: skin and fascia	1	Wounds without skin loss not over the fracture
	2	Wounds without skin loss over the fracture site
	3	Wounds with skin loss not over the fracture
	4	Wounds with skin loss over the fracture
	5	Circumferential wound with skin loss
Skeletal structures: bone and joints	1	Transverse/oblique fracture/butterfly fragment < 50% circumference
	2	Large butterfly fragment > 50% circumference
	3	Comminution/segmental fractures without bone loss
	4	Bone loss < 4 cm
	5	Bone loss > 4 cm
Functional tissues: musculotendinous (MT) and nerve units	1	Partial injury to MT unit
	2	Complete but reparable injury to MT units
	3	Irreparable injury to MT units/partial loss of a compartment/complete injury to the posterior tibial nerve
	4	Loss of one compartment of MT units
	5	Loss of two or more compartments/subtotal amputation
Comorbid conditions	2	Injury – debridement interval > 12 hours
	2	Sewage or organic contamination/farmyard injuries
	2	Aged > 65 years
	2	Drug-dependent diabetes mellitus/cardiorespiratory diseases leading to increased anesthetic risk
	2	Polytrauma involving chest or abdomen with injury severity score > 25/fat embolism
	2	Hypotension with systolic blood pressure < 90 mmHg at presentation
	2	Another major injury to the same limb/compartment syndrome
Total score		

Given this understanding, in healthcare settings with a scarcity of orthopaedic and plastic surgeons, the GHOISS holds substantial value for junior doctors in making decisions regarding patient transfers, and engaging in discussions with patients and their families about potential outcomes.^16^ This study aims to assess the applicability of the GHOISS to predict amputation versus limb salvage (in mangled limbs) in the context of Rwandan tertiary hospitals.

## Methods

This was a prospective cohort study conducted from March to September 2022 in two leading referral hospitals in Rwanda (Kigali University Teaching Hospital and Rwanda Military Hospital). We enrolled patients aged 18 years and above with open tibia fractures Gustilo and Anderson IIIA and IIIB after initial debridement, who were managed in two teaching hospitals in Kigali, Rwanda. Patients who underwent the initial debridement out of the selected study site, who were lost to follow-up or died during the follow-up period, or had a severe associated injury to the foot and ankle were excluded.

### Study procedure

Patients were received at the emergency departments (EDs) as per hospital protocol. The orthopaedic team reviewed the patients and classified the open fractures according to the Gustilo and Anderson classification. Patients who met the inclusion criteria and consented to the study’s participation were enrolled. They were admitted and debrided by the duty team (orthopaedic surgeon and orthopaedic surgery registrar) and their wounds were either confirmed or reclassified according to the Gustilo and Anderson classification, GHOISS. In-hospital information was filled in after debridement and at discharge by the patients. Outcome data were also collected at one month and six months’ follow-up at the outpatient department (OPD) on a data collection sheet. All data were kept in the study database.

### Data collection

Data were collected at three different times (admission to hospital, one month’s follow-up, and six months’ follow-up). Collected data included demographic data (age, sex, place of residence, and name of hospital) and clinical data (time and date of injury, time and date of hospital presentation, referral to another hospital or accident site, vitals at arrival, time of first antibiotic dose, time of surgical debridement, GHOISS, primarily amputated or salvaged, late amputation, date of discharge, length of hospital stay, need for soft-tissue reconstruction, and presence of surgical site infection – defined as none, superficial, or deep according to centre of disease). We subdivided our study participants into three groups according to GHOISS (Table I). Group I has a score of 1 to 13, Group II has a score of 14 to 16, and Group III has a score ≥ 17.

### General characteristics

This prospective cohort study enrolled 111 patients who sustained open tibia fractures which were managed at the two aforementioned public referral hospitals; the first hospital had 88 patients (79.3%) and the second hospital had 23 patients (20.7%). The male-to-female ratio was 3.44:1, and the left side was more frequently injured (n = 72 (64.8%)). The mean age at presentation was 34 years (18 to 80, SD 38.12). The majority of patients presented to EDs within 24 hours of injury (n = 85 (76.6%)). There was a significant delay to initial debridement, with a mean time of 82 hours (6 to 480, SD 79.17) and only 15 (13.5%) being debrided within the first 24 hours (Table II).

**Table II.** Patient demographic data.

Variable	Group I	Group II	Group III
Patients, n	97	11	3
Mean age, yrs (range)	37.95 (18 to 80)	40.36 (21 to 64)	45.66 (34 to 69)
**Sex, n**			
Female	24	0	1
Male	73	11	2
**Hospital, n**			
CHUK	75	8	3
RMH	22	3	0
**Injured side, n**			
Left	64	7	1
Right	33	4	2

Group I: GHOISS 1 to 13, Group II: GHOISS 14 to 16, Group III: GHOISS ≥ 17.

CHUK, Kigali University Teaching Hospital; GHOISS, Ganga Hospital Open Injury Severity Score; RMH, Rwanda Military Hospital.

### Statistical analysis

Data analysis was done using SPSS v. 27 (IBM, USA). Descriptive statistics (mean, range, frequency, rate) were used to analyze the demographic and clinical patient characteristics. The predictive values of the GHOISS in predicting the amputation or salvage were determined by calculating its sensitivity, specificity, and negative and positive predictive values. The positive test was a GHOISS ≥ 14, while the negative test was a GHOISS < 14; disease was present in the case of amputation and disease was negative in the case of salvage. Spearman’s rank correlation test was used to correlate GHOISS to surgical site infections, and the correlation between the GHOISS sub-score addressing skin and covering tissues to the need for reconstruction procedures. We also used an analysis of variance (ANOVA) test comparing groups of patients divided according to GHOISSs to their length of hospital stay. A p-value < 0.05 denoted a significant relationship.

## Results

### Clinical characteristics and outcomes

Patient presentation post-trauma to tertiary hospital was delayed, and there was a considerable delay in debridement in Groups I and II. Most of the patients in Groups II and III were classified as Gustilo and Anderson IIIB. The length of hospital stay was longer in Group II compared to the other groups. Almost all patients in Groups II and III were diagnosed with a fracture-related infection. Soft-tissue reconstruction was common in Groups I and II, whereas Group III patients were all amputated (Table III).

**Table III.** Patient clinical characteristics and outcomes.

Variable	Group I	Group II	Group III
Mean time to admission, hrs (SD)	30.25 (59.79)	20.36 (13.91)	16.33 (13.27)
Mean time to debridement, hrs (SD)	83.8 (77.75)	80 (98.61)	20 (12.12)
**Gustilo and Anderson, n**			
IIIA	64	1	0
IIIB	33	10	3
Amputation, n	4	7	3
Mean length of hospital stay, days (SD)	24.55 (25.75)	84.81 (53.12)	25.33 (4.04)
Infection, n	47	11	2
**Soft-tissue reconstruction, n**			
Yes	35	7	0
No	62	4	3

### Predictive values of GHOISS

When the GHOISS was 14 or higher, it correctly predicted ten patients who needed amputation, but incorrectly predicted amputation in four cases. This resulted in a 100% sensitivity and a 71.4% positive predictive value. Similarly, a GHOISS of less than 14 correctly predicted limb salvage in 97 patients, with a 96.03% specificity and 100% negative predictive value. The GHOISS demonstrated excellent accuracy in predicting amputation in patients with open tibia fractures (Table IV).

**Table IV.** Amputation prediction.

Ganga Hospital Score	Amputated	Not amputated
GHOISS ≥ 14	10	4
Predictive values	100%	71.4%
GHOISS < 13	Sensitivity 0	PPV 97
Predictive values	96.03% Specificity	100% NPV

NPV, negative predictive value; PPV, positive predictive value.

### Performance for GHOISS in predicting amputation

**Fig. 1:**
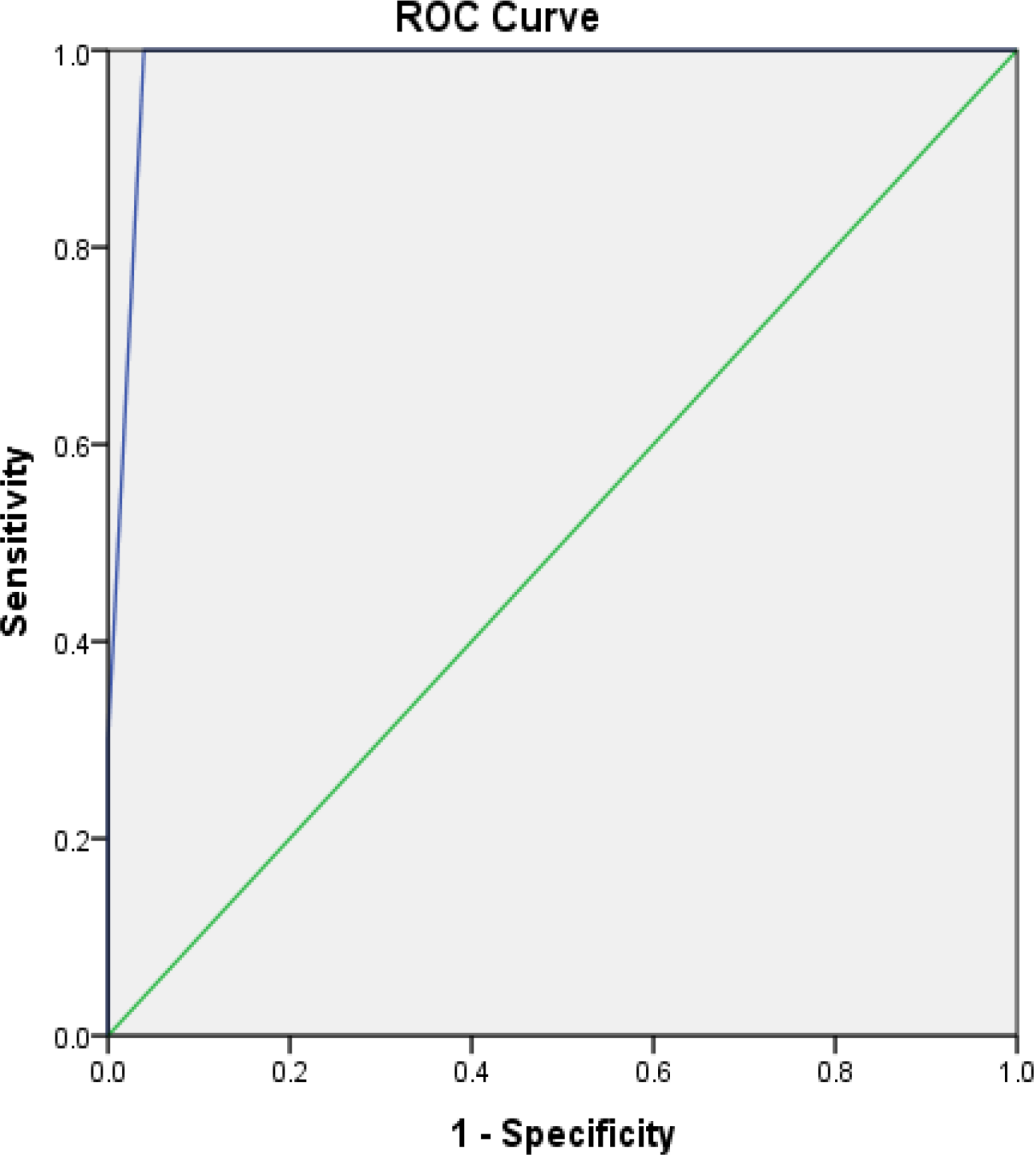
Receiver operating characteristic curve on amputation.

We plotted the receiver operating characteristic (ROC) curve for GHOISS above the diagonal line, indicating a high correlation between sensitivity and specificity. The area under the curve was 0.98, indicating excellent discriminatory ability (Figure 1).

### Length of hospital stay

Patients with a GHOISS of 1 to 13 had a wide range of stay lengths (3 to 120 days), with a mean stay of 24.55 days (SD 25.75). Patients with a GHOISS of 14 to 16 had longer stays (16 to 164 days), with a mean stay of 84.81 days (SD 53.12). Patients with a GHOISS score of 17 or higher had a more consistent range of stay lengths (23 to 30 days). Statistical analysis to compare the length of hospital stay between groups was conducted using ANOVA. The analysis revealed a significant difference in the mean lengths of hospital stay between the three groups: F (2,108) = 21.12; p < 0.001, which clinically implies that increasing total GHOISS is associated with increasing length of hospital stay. Table V shows the range, mean, and SD of hospital stay lengths for patients with different GHOISSs.

**Table V.** Ganga Hospital Open Injury Severity Score (GHOISS) and length of hospital stay.

GHOISS	Mean length of hospital stay, days (SD, range)
1 to 13	24.55 (25.75, 3 to 120)
14 to 16	84.81 (53.12, 16 to 164)
≥ 17	25.33 (4.04, 23 to 30)

### Soft-tissue reconstruction

We evaluated the correlation between the components of the GHOISS related to covering tissues and the necessity of soft-tissue reconstruction. Out of the 45 patients who had a covering tissue score of 3 or higher, 34 needed soft-tissue reconstruction. However, among the 66 patients with a score lower than 3, only four needed soft-tissue reconstruction (Table VI).

**Table VI.** Soft-tissue reconstruction requirement.

CTS	Reconstruction, n	No reconstruction, n	Correlation
1	3	0	*r* = 0.7
2	59	4	
3	4	2	
4	4	30	
5	3	2	

CTS, covering tissue score.

Statistical analysis using Spearman’s rank correlation test revealed a strong positive correlation between the GHOISS component scores and the need for soft-tissue reconstruction, with a correlation coefficient (Rs) of 0.7. This signifies that a higher score of covering tissue strongly correlates with the need for soft-tissue reconstruction (Table VI).

### Infection

The occurrence of infection in different groups was related to total GHOISS. Infections were classified as deep or superficial. Our data revealed an uneven distribution of infection between different groups, with more deep infection in Group III and more superficial infection in Group I (Table VII).

**Table VII.** Wound infections.

GHOISS	Wound infections			Correlation
	None	Superficial	Deep	
				*r* = 0.462
1 to 13	50	36	11	
14 to 16	0	1	10	
≥ 17	1	0	2	

GHOISS, Ganga Hospital Open Injury Severity Score.

Spearman’s rank correlation tests were conducted to examine the correlation between the GHOISS and the infection status of the wounds. The analysis shows a moderate positive correlation between the GHOISS and the infection status of patients’ wounds. The correlation coefficient was 0.462, indicating that as the severity of open tibia fractures increases, the infection status of the wounds also worsens (Table VII).

## Discussion

The general data characteristics (age, sex, injured side) of our study population were comparable to those reported in other similar studies. Fractures were most common in middle-aged patients, males, and those mostly injured on the left side. Conversely, there was a remarkable increase in delays to hospital presentation and initial debridement compared to other studies from HICs, which reported a mean time to definitive fixation of two days,^3^ and to a LIC study, where Hailu and Gebreyohanes^7^ reported that 85% of cases did not present to the hospital within two hours and more than 65% were not debrided within 24 hours.

The GHOISS demonstrated a remarkably high level of accuracy in predicting amputations at a score of 14 and above. It is noteworthy that our findings align with results found in several other studies, including a meta-analysis conducted by Ndlovu et al^19^ which revealed that GHOISS has a high sensitivity and specificity in predicting limb amputation at a score of 14 and higher, as well as the original GHOISS study by Rajasekaran et al,^18^ which reported a sensitivity of 98%, specificity of 100%, positive predictive value of 100%, and negative predictive value of 70%, and similar to the study by Ndlovu et al.^19^ In our study, the use of GHOISS could have helped in timely decision-making.

The study found that patients with a GHOISS of 14 to 16 (Group II) had significantly longer hospital stays compared to those in Groups I and III. This is significant, as open fractures, such as those of the tibia, are associated with extended treatment duration due to the intricate management of skeletal and soft-tissue injuries. The GHOISS effectively captures the complexity and severity of patients’ conditions, contributing to the current understanding of patient management in open fractures. Our results were comparable to those found by Rajasekaran and Sabapathy^12^ in 2007. However, our mean length of hospital stay was significantly higher in comparison to that reported by Parikh et al.^20^ We attribute this to a delay in soft-tissue reconstruction and amputation in borderline cases.

Our study found a high correlation between the covering tissue score (a sub-score of GHOISS) and the need for soft-tissue reconstruction. A score of 3 and above was associated with the need for soft-tissue reconstruction. It was also comparable to the results obtained by Madhuchandra et al,^21^ who reported similar results to those published initially by Rajasekaran et al.^12,16,18^ These findings are useful as they can help clinicians in the timely transfer of patients who are likely to need soft-tissue reconstruction to centres whose surgeons have the required skills, especially in limited-resource settings.

The infection rate was found to be high (54%) and moderately correlated with GHOISS. Our data results when compared to other were comparable to other published data, we found a significantly higher infection rate of 54%, as opposed to 16.7% published by Berner et al^3^ in a multicentre study in line with other multiple reported data.^12,16,18^ This high rate of infection can be linked to the significant delays in both presentation and management.

One limitation of this study is that we only gathered data from two centres, which makes it challenging to generalize the findings to the entire population.

In summary, the GHOISS has shown exceptional discriminative capabilities in predicting amputation likelihood and limb salvage in complex injuries. Its potential extends beyond amputation prediction to include related clinical outcomes. Its sub-score for skin and soft-tissue injuries predicts the need for soft-tissue reconstruction in open tibia fracture patients. Incorporating the GHOISS could enhance amputation determination precision and timeliness, potentially optimizing patient care and outcomes.

**Take home message**

- The Ganga Hospital Open Injury Severity Score has shown exceptional discriminative capabilities in predicting amputation likelihood and limb salvage in complex injuries.

## Data Availability

The data that support the findings for this study are available to other researchers from the corresponding author upon reasonable request.
